# A validated antibody panel for the characterization of tau post-translational modifications

**DOI:** 10.1186/s13024-017-0229-1

**Published:** 2017-11-21

**Authors:** Ebru Ercan, Sameh Eid, Christian Weber, Alexandra Kowalski, Maria Bichmann, Annika Behrendt, Frank Matthes, Sybille Krauss, Peter Reinhardt, Simone Fulle, Dagmar E. Ehrnhoefer

**Affiliations:** 1 BioMed X Innovation Center, Im Neuenheimer Feld 515, 69120 Heidelberg, Germany; 2 AbbVie GmbH&Co KG, Knollstraße 50, 67061 Ludwigshafen am Rhein, Germany; 3 Institute for Molecular and Cell Biology, Mannheim University of Applied Sciences, Paul-Wittsack-Straße 10, 68163 Mannheim, Germany; 4 German Center for Neurodegenerative Diseases (DZNE), Sigmund-Freud-Straße 27, 53127 Bonn, Germany

**Keywords:** Tau, Post-translational modification, Antibody validation, Alzheimer’s disease

## Abstract

**Background:**

Tau is a microtubule-binding protein, which is subject to various post-translational modifications (PTMs) including phosphorylation, methylation, acetylation, glycosylation, nitration, sumoylation and truncation. Aberrant PTMs such as hyperphosphorylation result in tau aggregation and the formation of neurofibrillary tangles, which are a hallmark of Alzheimer’s disease (AD). In order to study the importance of PTMs on tau function, antibodies raised against specific modification sites are widely used. However, quality control of these antibodies is lacking and their specificity for particular modifications is often unclear.

**Methods:**

In this study, we first designed an online tool called ‘TauPTM’, which enables the visualization of PTMs and their interactions on human tau. Using TauPTM, we next searched for commercially available antibodies against tau PTMs and characterized their specificity by peptide array, immunoblotting, electrochemiluminescence ELISA and immunofluorescence technologies.

**Results:**

We demonstrate that commercially available antibodies can show a significant lack of specificity, and PTM-specific antibodies in particular often recognize non-modified versions of the protein. In addition, detection may be hindered by other PTMs in close vicinity, complicating the interpretation of results. Finally, we compiled a panel of specific antibodies and show that they are useful to detect PTM-modified endogenous tau in hiPSC-derived neurons and mouse brains.

**Conclusion:**

This study has created a platform to reliably and robustly detect changes in localization and abundance of post-translationally modified tau in health and disease. A web-based version of TauPTM is fully available at http://www.tauptm.org.

## Background

The formation of neurofibrillary tangles due to aggregation of the microtubule associated protein tau is at the center of multiple neurodegenerative disorders, including progressive supranuclear palsy (PSP), frontotemporal dementia and parkinsonism linked to chromosome 17 (FTDP-17), corticobasal degeneration, Pick’s disease, Alzheimer’s disease (AD) and others [1]. Under physiological conditions, tau binds to and stabilizes microtubules via its C-terminal domain [2, 3]. It is a natively unfolded, highly hydrophilic protein and is stable under high temperatures and acidic conditions [4]. In the adult human brain, six different isoforms can be distinguished based on the presence or absence of repeat domain 2 (3R or 4R) and the presence of 0, 1 or 2 N-terminal inserts [5]. While tau mutations are linked to FTDP-17 and tau polymorphisms are risk factors for PSP and corticobasal degeneration, no tau mutations have so far been linked to AD [1]. However, tau receives extensive post-translational modifications (PTMs) such as phosphorylation, acetylation, nitration, methylation, glycosylation, deamination, ubiquitination and sumoylation. Many of these PTMs are altered in AD and may contribute to tau dysfunction and pathology (reviewed in [6]).

In order to study the effects of PTMs on tau function, antibodies raised against specific modification sites are commonly used. However, due to limitations in available tools and resources, the majority of such antibody-based approaches lack appropriate quality control experiments. The most common problems with antibodies include cross-reactivity with nonspecific targets, variability from batch to batch and the use of wrong experimental systems [7]. To evaluate cross-reactivity, antibodies should be tested on a sample, which does not contain the target protein itself (knockout control) and/or the specific modification in the case of PTM-specific antibodies. As epitopes may furthermore be influenced by protein conformation and thus react differently with native versus denatured samples, the specificity and efficiency of an antibody may vary depending on the technique employed. Additionally, proteins heavily modified by PTMs may escape antibody detection due to steric hindrance and thus lead to false-negative results.

In this study, we first identified tau PTMs that have been found in primary human tissue through a thorough database and literature search. We established an online tool for the visualization of these PTMs (called ‘TauPTM’) with access links to the original literature. We then explored commercially available antibodies raised against these tau modifications and tested their specificity for PTM-modified tau as well as for tau in general using peptide arrays and immunoblotting. We furthermore included a panel of commercially available antibodies detecting all tau species to generate a comprehensive toolbox for the study of tau PTMs. Using these tools, we demonstrate the presence of a large variety of tau PTMs in transgenic mice expressing human tau protein (hTg-Tau model, [8]) and evaluate the subcellular localization of PTM-modified tau species in iPSC-derived neurons.

Taken together, our antibody validation data and the TauPTM online tool thus provide a valuable resource and platform for researchers studying tau.

## Methods

### Peptide Array

The peptide array was prepared by Peptides and Elephants GmbH (Berlin, Germany). Synthesized peptides with or without specified modifications were quality controlled by HPLC and MS and were attached C-terminally on trioxatridecanediamine (TOTD) membranes. The membranes were treated 10 min with methanol and washed three times with 1X TBS to activate. After 2 h of blocking with 4% BSA in 1X TBST (1X TBS, 0.05% Tween-20), overnight incubation was performed in blocking solution with 1 μg/ml primary antibody at 4 °C. The next day, the membranes were washed three times with 1X TBST and secondary antibody incubations (1:20,000, IRDye Donkey anti-mouse 800 and IRDye Goat anti-rabbit 680) were done in blocking solution for 2 h at room temperature. The membranes were then washed three times with 1X TBST and once with 1X TBS and developed with a Li-Cor Odyssey CLx Imaging station.

### Mouse brain and human entorhinal cortex lysate preparation and immunoblotting

Mice were maintained in a temperature-controlled room (22 °C, 60% air humidity) with a light/dark cycle of 12 h/12 h and had access to food and water ad libitum. Animals were sacrificed at nine months of age by cervical dislocation and brains were snap-frozen in liquid nitrogen and stored frozen at −80 °C until lysis. All procedures were in compliance with German Animal Protection Law and were approved by the competent authorities (Landesamt für Naturschutz und Verbraucherschutz Nordrhein-Westfalen; AZ 87–51.04.2011.A049/01). Anonymized human post-mortem tissue was obtained from the London Neurodegenerative Diseases Brain Bank, a member of the Brains for Dementia Research Network. Tissue donor characteristics were as follows: Donor BBN_18399: male, 77 years, 11 h post-mortem interval, cause of death: metastatic prostate cancer. Donor BBN_24243: female, 69 years, 48 h post-mortem interval, cause of death: advanced pancreatic cancer. Donor BBN_13802: male, 74 years, 22.5 h post-mortem interval, cause of death: respiration failure; cancer.

Lysates from hTg-Tau and Tau-KO mice and human entorhinal cortices were prepared by dounce homogenizer in 350 μl of lysis buffer containing 150 mM NaCl, 20 mM Tris pH 7.5, 1 mM EDTA, 1 mM EGTA, 1% Triton-X100 and protease, phosphatase, demethylase (500 μM IOX1, 2 μM Daminozide, 10 μM Paragyline Hydrochloride), deacetylase (10 μM Trichostatin A, 5 mM Nicotinamide), O-GlcNAcase (1 μM Thiamet-G) inhibitors. The protein concentration was measured by BCA assay according to manufacturer’s instructions (BioRad). 20 μg of protein, which was boiled at 95 °C for 5 min in sample buffer, was separated on an SDS-gel and was immunoblotted on PVDF membranes. The membranes were blocked with 5% BSA in 1X TBST and primary antibody (1:1000) incubations were performed in blocking solution overnight at 4 °C. The next day, secondary antibody incubations (1:20,000, IRDye Donkey anti-mouse 800 and IRDye Goat anti-rabbit 680) were done in blocking solution for 2 h at room temperature. The membranes were then washed three times with 1X TBST and one with 1X TBS and developed on a Li-Cor Odyssey CLx imaging station. In the case of no or low signal detection, the immunoblotting was repeated with 50 μg of protein and primary antibodies diluted 1:500. Actin (Sigma-Aldrich, Cat No. A2103) was used as a loading control.

### Purification of recombinant tau and caspase 3 cleavage

The tau 2N4R isoform (aa1–441) and the truncated tau (aa1–421) was recombinantly expressed in *E. coli* strain BL21(DE3) (Sigma-Aldrich, Cat. No. CMC0014) using pET19b as expression vector. Protein purification was performed as previously described with modifications described below [9]. Briefly, the frozen *E.coli* pellets were suspended in sodium-phosphate buffer (50 mM NaPi, 1 mM EGTA, 1 mM DTT, pH 7) and incubated for 30 min at 100 °C. After centrifugation (12 000×g, 4 °C, 15 min) the filtrated supernatant was purified with liquid chromatography using a HiTrap™ IEX HP column (Fisher Scientific, Cat. No. 11748508): The column was washed with sodium-phosphate buffer (50 mM NaPi, 1 mM EGTA, 1 mM DTT, pH 7) and the protein was eluted using a gradient of 0–300 mM NaCl in sodium-phosphate buffer. The column fractions containing pure protein, as determined by gel electrophoresis, were pooled and concentrated with Amicon centrifugal filter units (Merck Millipore, Cat. No. UFC203024).

For the caspase digestion, 3 μg of purified tau 2N4R protein (tau 1–441) were mixed with caspase 3 enzyme (Enzo Life Science, Cat. No. BML-AK010–0001) in caspase buffer (100 mM HEPES pH 7,4, 200 mM NaCl, 0,2% CHAPS, 2 mM EDTA, 20% Glycerol, 10 mM DTT). This mixture was incubated for 1 h at 37 °C. The digestion was analyzed by Western blot.

### Electrochemiluminescence ELISA

Meso Scale Discovery (MSD) Gold Streptavidin small-spot 96-well plates were blocked with 5% (*w*/*v*) Blocker A solution in Tris Wash Buffer (50 mM Tris-HCl pH 7.5, 150 mM NaCl and 0.02% Tween-20). Plates were sealed and allowed to block for 1 h at room temperature (RT) on a plate shaker. The plates were then washed three times with Tris Wash Buffer and coated with 30 μL of biotinylated antibody diluted in 1% Blocker A solution. The biotinylation of the antibodies were performed according to the manufacturer’s instructions (EZ-Link Sulfo-NHS-Biotin, Cat No. 21217, Thermo Scientific). Before biotinylation, BSA was removed with the Melon Gel IgG Purification Kit (Cat. No 45212, Thermo Scientific) if necessary. After incubating the plates for 1 h at RT on a plate shaker, they were washed three times with Tris Wash Buffer. 5 μg of protein lysates (diluted in 50 μl) were added and incubated for 1 h at RT on a plate shaker. Plates were washed three times with Tris Wash Buffer to get rid of unbound lysates and were incubated with 25 μl of 0.5 μg/ml detection antibody (Tau12 labeled with MSD Sulfo-Tag-NHS-Ester, Cat. No: R31AA, Meso Scale Discovery) diluted in 1% Blocker A solution for 1 h at RT on a plate shaker. The plates were then washed three times with Tris Wash Buffer and 150 μl of 2X Read Buffer (Cat. No. R92TC, Meso Scale Discovery) were added 5 min before the signal was measured on a Meso Scale Discovery Quickplex platform.

### Generation of hiPSC-derived neurons

hiPSCs generated from healthy donor fibroblasts reprogrammed with Sendai Virus were obtained through the StemBANCC consortium. Cells were cultured in mTeSR1 medium (Stemcell Technologies) on Matrigel (Corning). To generate hiPSC – derived neurons, hiPSCs were first differentiated into neural progenitor cells (NPCs) according to [10] with minor modifications. Briefly, NPCs were induced from iPSCs in N2/B27 medium (consisting of Advanced DMEM/F12:Neurobasal (1:1), 1X N2, 1X B27, 1% L-Glutamine, 1% Pen/Strep (all Thermo), 5 μg/mL BSA (Sigma)) supplemented with 10 ng/mL hLIF (Millipore), 4 μM CHIR99021, 3 μM SB431542 (both Tocris), 0.1 μM Compound E (Calbiochem) for 11 days. NPCs were further cultured on Matrigel - coated plates in N2/B27 medium supplemented with hLIF, CHIR99021, SB431542, with additional 10 ng/ml FGF2 (R&D).

For differentiation into neurons, NPCs were seeded at 8 × 10^5^ cells / cm^2^ on laminin (Sigma) coated plates in N2/B27 without supplements and maintained for 11 days with medium change every 2–3 days. For the final maturation, hiPSC – derived neurons were dissociated using Accutase (Thermo) and re-plated at the same density on poly-D-lysine coated pre-coated plates (Greiner), coated with laminin in maturation medium (N2/B27 supplemented with 1 mM dbcAMP, 200 nM ascorbic acid (both Sigma), 5 μg/ml laminin, 2 ng/ml BDNF (Peprotech) and 2 ng/ml GDNF (R&D)). Medium changes were performed twice a week for additional 4 weeks.

### Immunofluorescence

hiPSC-derived neurons in 96-well plates were fixed with 4% Paraformaldehyde and permeabilized with 0.1% Triton-X100 in 1X TBS for 10 min at RT. Cells were then washed three times with 1X TBS and blocked with 5% BSA in 1X TBS for 1 h at RT. Primary antibody incubation (1:50) was performed in 2.5% BSA in 1X TBS overnight at 4 °C. The primary antibody dilution was decreased to 1:25 when no signal was detected. The next day, the cells were washed three times with 1X TBS and secondary antibody (Alexa Fluor goat anti-mouse 488 and Alexa Fluor goat anti-rabbit 488) incubation was performed in 2.5% BSA in 1X TBS for 2 h at RT. Cells were washed two times with 1X TBS and incubated with 1X TBS containing Hoechst (1:20,000) for 10 min at RT and then washed once with 1X TBS and cells were kept in 1X TBS for microscopy. Microscopy was performed with Zeiss inverted fluorescence microscope with 20X objective and images were processed in ImageJ.

## Results

### Visualizing the post-translational modification (PTM) sites on human tau: TauPTM tool

Tau PTMs have been studied extensively in the context of AD as well as other neurodegenerative diseases [6]. Many studies using different experimental approaches and model systems show that PTMs affect the function of tau by modulating its binding to microtubules, its degradation, subcellular localization and secretion [6]. However, the excessive information coming from human samples, different model systems and experimental approaches makes it difficult to summarize current knowledge about tau modifications in human brain. Additionally, researchers are becoming increasingly aware of interplay between different PTMs, in particular on heavily modified proteins such as tau. PTMs can be mutually exclusive (targeting the same amino acid), or prevent or promote subsequent modifications through conformational changes [11]. In order to provide an overview of the current state of knowledge of the tau PTM landscape and also facilitate the visualization of potential or proven interactions between PTMs, we designed an interactive tool, which comprises all modifications that have been shown in human brain so far. Initially, tau PTMs were retrieved from the dbPTM database, which combines experimentally validated PTM sites from a number of resources including the Human Protein Reference Database (HPRD 9.0), the Phospho.ELM database (PhosphoELM.10011), PhosphoSitePlus (Phosphositeplus.1010730), UniProtKB/Swiss-Prot (Phosphositeplus.1010730), and SysPTM (SysPTM 1.1). We used the Uniprot ID: P10636 to query dbPTM for microtubule-associated protein tau (MAPT) entries, and mapped the modified residue positions to the PNS-tau isoform and then to the respective residue in the 2N4R tau isoform. We then verified the entries and added recent information by manual literature search. While curating the data we applied these rules: 1) The modification has been shown in human brain samples with mass spectrometry and/or immunohistochemistry. 2) The modifications shown in other model systems such as mouse or cultured cells were only added as supporting information in the literature section. These stringent criteria prevent the inclusion of PTMs that may have arisen as artifacts of the model system with no relevance for human brain. The current version of TauPTM contains 53 phosphorylation, 8 acetylation, 2 deamidation, 10 methylation, 5 dimethylation, 4 nitration, 4 ubiquitination and 6 proteolytic cleavage sites present on human tau (www.tauptm.org). In addition to the single PTMs of tau, we entered known cross-reactions between modifications such as the finding that acetylation at K280 prevents the phosphorylation at T212 and S214, whereas it promotes the phosphorylation at S262. These cross-reactions are visualized by color-codes on the respective amino acids as well as in a table format, allowing for the fast visual inspection of potential known interactions for single PTMs.

### Validation of post-translational modification-specific tau antibodies with peptide arrays

**Fig. 1:**
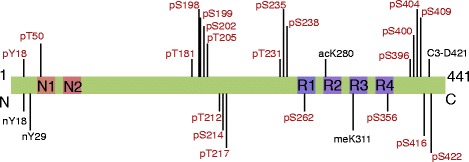
Scheme of tau (2N4R) with PTM sites that are covered by commercially available antibodies. Phosphorylated residues are labeled in red, other modifications in black. N1 and N2: N-terminal inserts 1 and 2, respectively. R1, R2, R3, and R4: Repeat domains 1–4. pY: phospho-tyrosine, pT: phospho-threonine, pS: phospho-serine, nY: nitrated tyrosine, acK: acetylated lysine, meK: dimethylated lysine, C3-D: caspase-3 cleaved aspartic acid

We next used our TauPTM tool to search for commercially available antibodies raised against the single modifications. We found several antibodies covering 26 different modification sites (Fig. 1), approximately one third of all known human tau PTMs are therefore covered by commercially available antibodies. To test antibody specificities for PTM-tau over non-modified tau, we synthesized 15 amino acid-long peptides derived from the human tau sequence (2N4R isoform) with either no, single or multiple PTMs on a membrane. Since antibody epitopes may include amino acids N- or C-terminal of the detected PTM, we designed at least two peptides for each antibody, with the modified amino acid moved by five positions.

We tested a total of 36 nitration-, acetylation-, dimethylation- and phosphorylation-specific antibodies, focusing only on antibodies detecting specific single modifications rather than conformation specific antibodies which require more than one modification and a specific conformation/aggregation state of tau oligomers, as these conformational epitopes cannot be mimicked with synthesized peptides.

**Fig. 2:**
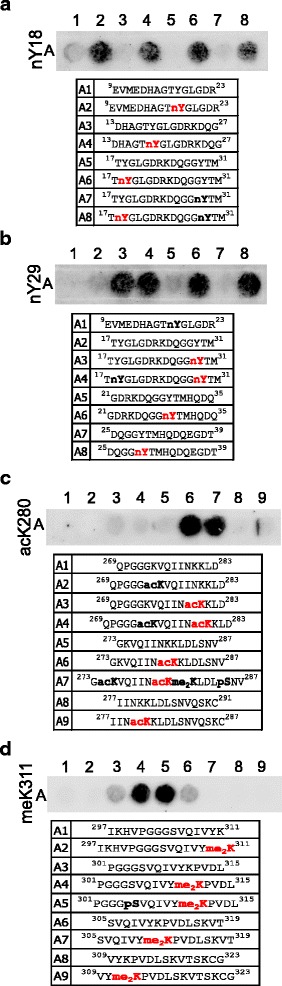
Peptide arrays validate the specificity of antibodies detecting non-phospho-PTMs on tau. **a** nY18, **b** nY29, **c** acK280 and **d** meK311 antibodies demonstrated specific detection of the respective modifications on a peptide array. While all antibodies are specific, the detection of acK280 (**c**) and meK311 (**d**) depends on the surrounding peptide sequence. The sequence and modification profile for peptides immobilized in each spot are shown below the blots. The modification targeted by the antibody is shown in bold red, off-target modifications are shown in bold black

Among the antibodies detecting nitrated, acetylated and dimethylated tau (Fig. 2), those raised against nitration on Y18 and Y29 (Fig. 2a, b), acetylated K280 (Fig. 2c) and dimethylated K311 (Fig. 2d) showed high specificity for their respective modifications with no detection of non-modified peptides. Furthermore, their binding was not affected by PTMs in the surrounding tau sequence, an important advantage when analyzing heavily modified protein samples.

**Fig. 3:**
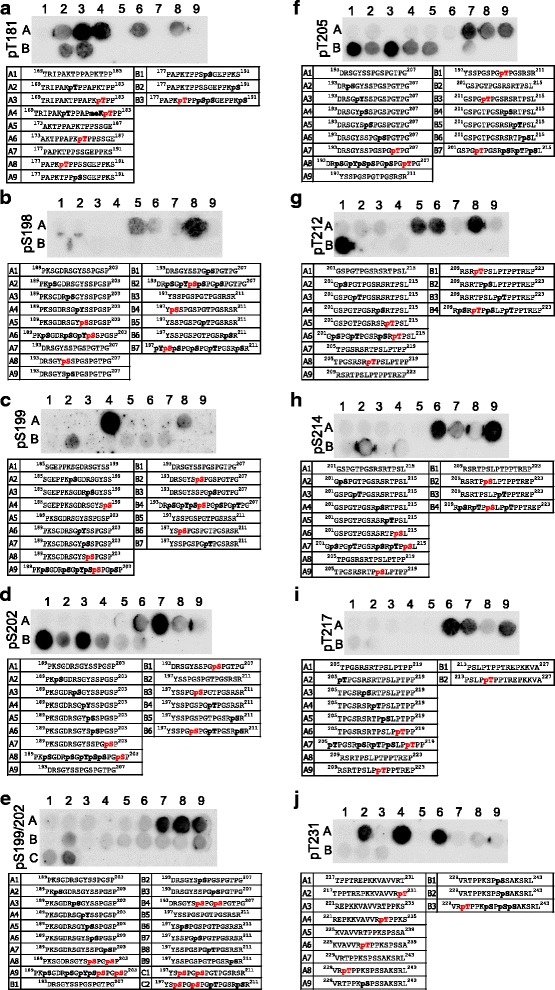
Peptide arrays validate the specificity of phospho-tau antibodies covering aa 181–231. **a** pT181, **b** pS198, **c** pS199, **d** pS202, **e** pS199/202, **f** pT205, **g** pT212, **h** pS214, **i** pT217 and **j** pT231 antibodies demonstrated specific detection of the respective modifications on a peptide array. While all antibodies are specific, the detection of pS198 (**b**), pS199 (**c**), pS202 (**d**) and pS214 (**h**) can be inhibited by additional phosphorylation events in the surrounding peptide sequence. The antibodies against pS202 (**d**), pS199/202 (**e**) and pT205 (**f**) also detect off-target modifications. The sequence and modification profile for peptides immobilized in each spot are shown below the blots. The modification targeted by the antibody is shown in red, off-target modifications are shown in bold black

**Fig. 4:**
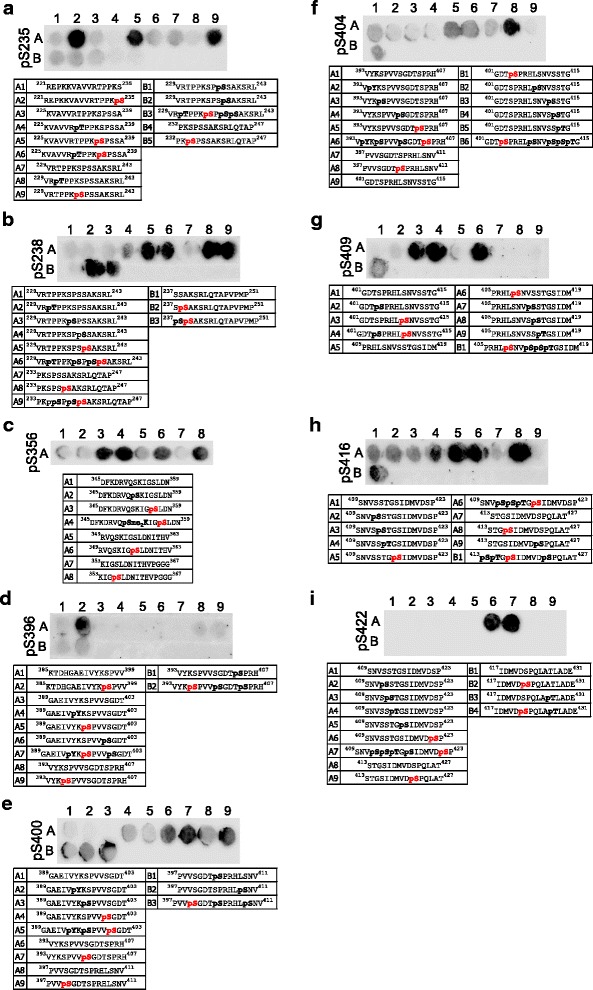
Peptide arrays validate the specificity of phospho-tau antibodies covering aa 235–422. **a** pS235, **b** pS238, **c** pS356, **d** pS396, **e** pS400, **f** pS404, **g** pS409, **h** pS416 and **i** pS422 antibodies demonstrated specific detection of the respective modifications on a peptide array. While all antibodies are specific, the detection of pS235 (**a**), pS396 (**d**) and pS400 (**e**) can be inhibited by additional phosphorylation events in the surrounding peptide sequence. The sequence and modification profile for peptides immobilized in each spot are shown below the blots. The modification targeted by the antibody is shown in red, off-target modifications are shown in bold black

Next, we tested commercially available phosphorylation-specific tau antibodies. As phosphorylation is a major PTM on tau and hyperphosphorylation is linked to tauopathies, a plethora of antibodies is available. We tested 32 phospho-tau antibodies raised against 22 phosphorylation sites that have been reported in human brain. All antibodies shown in Figs. 3 and 4 were specific for their respective phosphorylation site, however, for 7 out of 19 their binding was impaired by phosphorylation events at neighboring sites. For instance, binding of the antibody against phosphorylated S198, while highly specific for the modification, was hindered by other phosphorylation events in close vicinity (Fig. 3b).

**Fig. 5:**
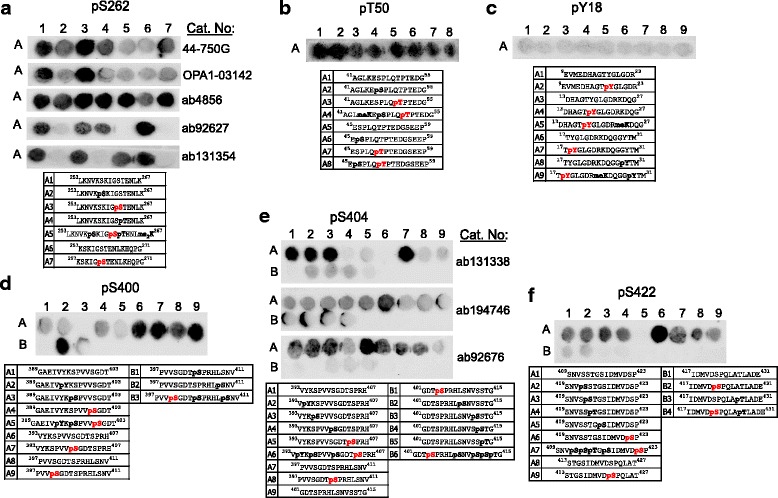
Peptide arrays demonstrate specificity issues for multiple phospho-tau antibodies. **a** Five different antibodies raised against pS262 detected the non-modified tau peptide A1. **b** The only available pT50 antibody detected non-modified peptides as well as those containing different modifications. **c** No signal was detected with the pY18 antibody for all peptides tested, suggesting that it may require a conformational epitope. **d** The only available pS400 antibody detected non-modified peptides A6 and A8. **e** All three different pS404 antibodies tested detected the non-modified peptides A1 and A7. **f** The only available antibody against pS422 detected the non-modified peptides A1 and A8 and also off-target modifications. Antibodies shown in (**a**, **b**, **d**, **e** and **f**) were discarded from the panel

Interestingly, for three antibodies (pT205, pT212 and pS409), only phosphorylation events C-terminal of the specific PTM site interfered with detection (Fig. 3f and g, Fig. 4g). Overall, however, this finding is concerning, as tau is most often modified at multiple sites at the same time and phosphorylation events may thus block antibody detection. Moreover, the antibodies raised against pS202, pS199/202, pT205, pS416 and pS422 detected other neighboring phosphorylation sites in addition to their specific residues (Figs. 3d-f, Fig. 4h and Fig. 5f).

Furthermore, we found a number of antibodies that, despite being sold as specific for phospho-tau at particular sites, did not distinguish between modified and non-modified versions of tau-derived peptides. Five different antibodies against phosphorylation at site Ser262 (pS262) gave very strong signals with non-modified peptides (Fig. 5a), and we were unable to identify one with sufficient specificity. Similarly, the only commercially available antibody against pT50 we are aware of did not meet our specificity criteria (Fig. 5b). For sites pS400, pS404 and pS422, we had to test several antibodies to identify one that performed well. Three out of four antibodies tested for pS404, and one out of two for each pS400 and pS422 showed strong binding to bare peptides or peptides modified at different sites (Fig. 5d-f).

Interestingly, only the antibody against the pY18 site showed no signal on the array (Fig. 5c). As the antibody was raised against a tau peptide spanning aa12–24, which is covered by our array, it is unlikely that its epitope is outside of the region covered in our experiment. However, it is possible that conformational changes caused by the PTM, which are not recapitulated on the array, are necessary for detection.

### Validation of tau antibodies with mouse brain lysates

**Fig. 6:**
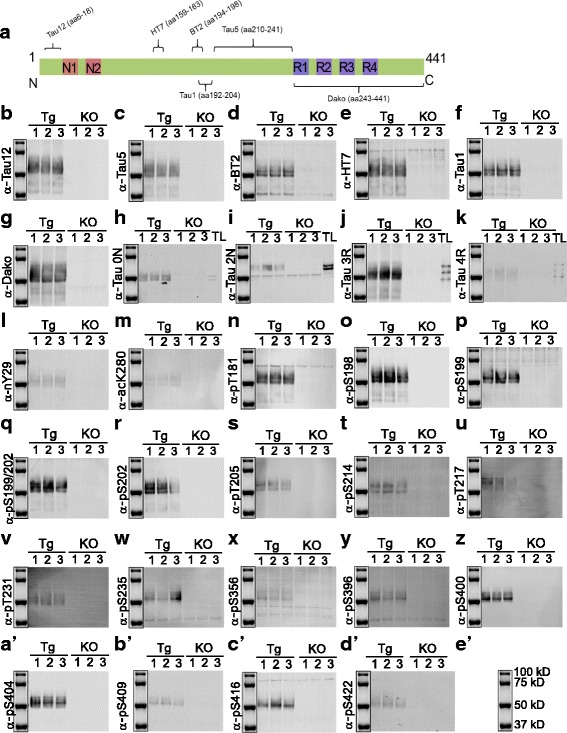
Immunoblotting using hTg-Tau and Tau-KO brain lysates confirms the specificity for tau for a panel of antibodies. **a**) Scheme of tau (2N4R) showing the epitopes of the Tau12, Tau5, Tau1, HT7, BT2 and Dako antibodies. **b**) - **d’**) Immunoblots demonstrate strong reactivity of all antibodies with tau bands between 40 and 60 kD. Minor non-specific bands present in Tau-KO brain lysates were detected in **d**), **e**), **i**), **h**), **p**), **x**), **y**) and **c**’). Isoform-specific antibodies in **h**), **i**), **j**) and **k**) were validated using a tau ladder with all 6 recombinant human tau isoforms. kDa sizes for the marker are given in **e’**). Tg: hTg-Tau mouse, KO: Tau-KO mouse brain lysate, TL: recombinant tau ladder

Having determined the specificity of antibodies for different PTM-modified versions of tau, we went on to assess non-specific signals from brain lysates by immunoblotting. In this assay, we included a panel of widely used, commercially available pan-tau antibodies (Tau1, Tau5, Tau12, BT2, HT7, Dako-Tau), which have various epitopes spanning the entire tau sequence (Fig. 6a), isoform-specific antibodies (0 N, 1 N, 2 N, 3R, 4R) and an antibody against Caspase 3-cleaved tau at D421 (Fig. 1, C3-D421). In order to show the specificity of the antibodies for tau, we used cerebral lysates from nine-month-old hTg-Tau mice expressing all six human tau isoforms on a mouse tau knockout background [8], and the corresponding Tau-KO mouse [12]. Amongst all pan-tau antibodies tested, only BT2 and HT7 showed nonspecific signals around 35 kD and 75 kD, respectively, as these bands were detected in Tau-KO samples (Fig. 6d, e).

**Fig. 7:**
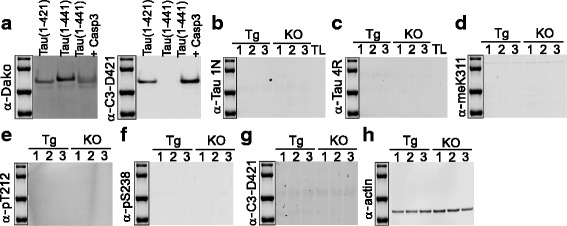
A number of antibodies failed to detect tau in hTg-Tau brain lysates. **a** Immunoblotting with recombinant tau (full length (1–441), truncated (1–421) and caspase 3 cleaved ((1–441) + Casp3) shows that all tau forms can be detected with a pan-tau antibody (Dako, left), whereas the C3-D421 antibody only detects the truncated and the caspase 3 cleaved form of tau (right). **b** and **c** Tau isoform-specific antibodies failed to detect tau in hTg-Tau brain lysates as well as the recombinant protein in the tau ladder. **d**, **e**, **f** and **g** No signal was detected by the meK311, pT212, pS238 and C3-D421 antibodies in hTg-Tau mouse brains, possibly due to the low abundance of these modifications. **h** Actin demonstrates equal loading for all samples. Tg: hTg-Tau mouse, KO: Tau-KO mouse brain lysate, TL: recombinant tau ladder

In order to test the specificity of the C3-D421 antibody, we used recombinant tau truncated at D421 (tau 1–421) as well as full length tau cleaved with caspase 3 in vitro (tau 1–441 + Casp 3). While all tau forms (full length, truncated and cleaved) were detected with the Dako antibody against total tau (Fig. 7a, left), we observed that C3-D421 antibody specifically detects tau 1–421, both expressed as a truncated protein and generated through caspase 3 cleavage in vitro (tau 1–441 + Casp3) (Fig. 7a, right).

To test the specificity of isoform specific antibodies, we used a commercially available tau ladder, which includes all six isoforms expressed in human brain. 1 N and one of the tested 4R antibodies were found to be nonspecific (Fig. 7b+c), whereas 0 N, 2 N, 3R and another 4R antibody only detected their corresponding isoforms. However, the 2 N antibody had an additional nonspecific signal around 50 kD as detected in Tau-KO samples (Fig. 6i). Most of the post-translational modification specific antibodies, which were validated by peptide arrays, showed specific binding to tau except pT181, pT199, pS356, pS396 and pS416. These antibodies bind to other proteins in Tau-KO samples (Fig. 6). The meK311, pS217, pS238, and C3-D421 specific antibodies did not give any signal (Fig. 7d-g) although the experiment was repeated multiple times with higher amounts of lysates and higher concentrations of the antibodies. Taken together, these results suggest that the meK311, pS217, pS238 and C3-D421 modifications either do not exist or only exist at nondetectable levels in the mouse brains.

We summarized the peptide array and immunoblotting results in Table 1, which shows the source of the antibodies and how they performed in both assays. When we classified the antibodies according to peptide array results we categorized them as very good, good, and average. If the antibody detected an additional modification site, it was classified ‘average’, and if its detection was hindered by other neighboring modifications as ‘good’. The antibodies classified as ‘very good’ showed no non-specific binding and their detection was not hindered by other modifications. The antibodies, which gave strong signals with non-modified peptides (Fig. 5), were discarded.

**Table 1:**
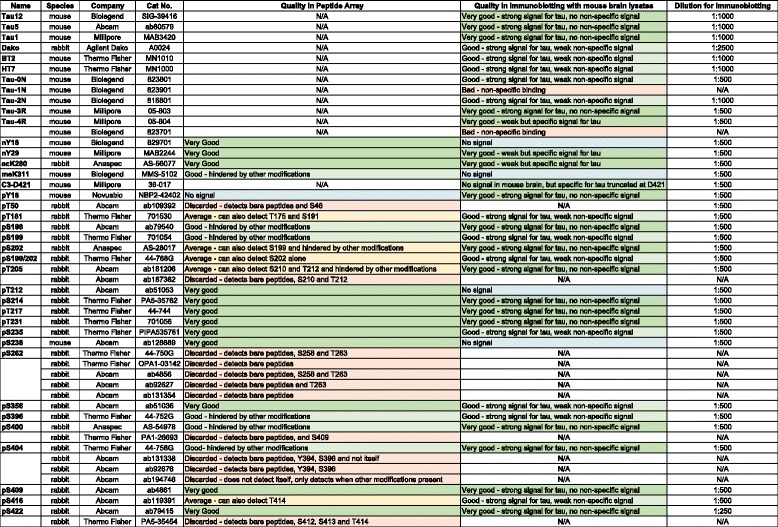
Quality of tau antibodies tested by peptide arrays and immunoblotting

Summarizes the quality of the pan-tau and PTM-specific tau antibodies, which are tested by peptide arrays (Figs. 2, 3, 4 and 5) and immunoblotting (Figs. 6 and 7). N/A: not assessed

Green: very good, light green: good, yellow: average, red: discarded, blue: no signal

For immunoblotting, antibodies that gave strong or weak but specific signal to tau were classified as ‘very good’. Antibodies detecting weak non-specific bands in tau-KO samples were classified as ‘good’. If the antibody was not specific to their intended target, such as 1 N and one of the 4R antibodies, they were classified as ‘bad’. If we could not detect any signal, we classified the antibody as ‘no signal’. Importantly, this last category does not reflect on the antibody quality, but rather on the absence of the analyte in hTg-Tau brain samples.

### Analysis of PTM-modified tau in human brain lysates

**Fig. 8:**
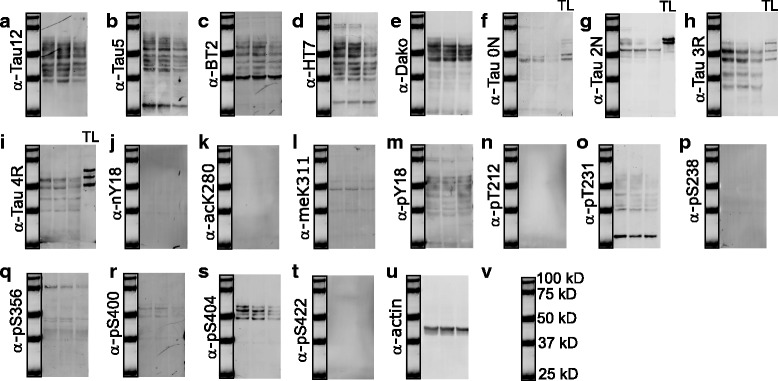
Human entorhinal cortex (EC) lysates show many but not all tau PTMs. **a**-**e** Immunoblots demonstrate strong reactivity of all pan-tau antibodies with human EC (*n* = 3). **f**-**i** detection with isoform specific tau antibodies reveals the presence of multiple isoforms of tau. TL: recombinant tau ladder. **j**-**t** PTM-specific antibodies demonstrate reactivity except for **j** nY18, **k** AcK280, **n** pT212, **p** pS238 and **t** pS422. **u** Immunoblotting with actin for loading control shows similar loading for three different EC lysates (n = 3). **v** kDa sizes for the marker

Next, we tested our validated antibodies on human entorhinal cortex lysates to confirm their suitability for the investigation of tau PTMs in patient samples (Fig. 8). We tested a total of 20 antibodies consisting of five pan-tau and 15 PTM-specific tau antibodies. Total tau antibodies with epitopes at the N-terminus or in the central region detected full-length tau isoforms, ranging from approximately 45–70 kDa on the gel, as well as shorter, presumably truncated forms (Fig. 8a-d). The Dako antibody on the other hand was raised against a C-terminal epitope (aa 243–441) and does not detect these fragments, suggesting they are C-terminally truncated (Fig. 8e). The isoform-specific antibodies raised against the repeat region further show that this epitope is still intact in some tau fragments ranging from approximately 35-40 kDa (Fig. 8h and i).

Western blotting with PTM-specific antibodies revealed that meK311, pY18, pT231, pS356, pS400 and pS404 are present in our samples (Fig. 8l–s), while nY18, acK280, pT212, pS238, and pS422 were not detectable, even when the amount of protein loaded on the gel was increased (Fig. 8j–t). This suggests that either these tau modifications do not exist or are below detection level in our human brain samples.

### Quantitative assessment of tau PTMs by electrochemiluminescence ELISA

**Fig. 9:**
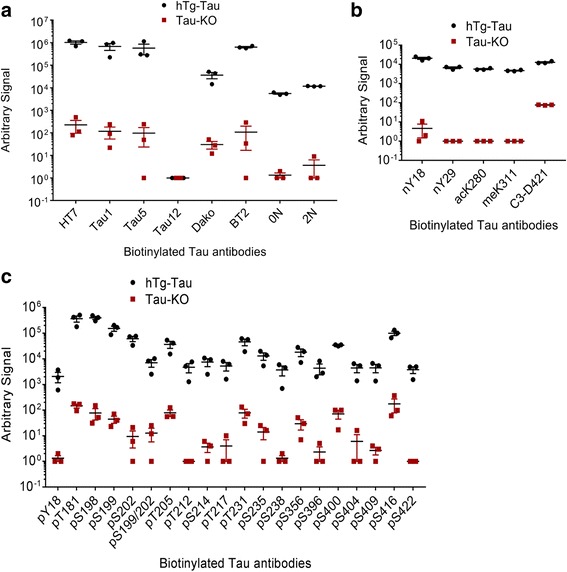
An electrochemiluminescence ELISA detects tau PTMs in hTg-Tau mouse brain lysates. Sandwich ELISA assays using biotinylated antibodies for capture and sulfo-tagged Tau-12 antibody for detection demonstrate strong and specific signals for all antibodies in the panel. **a** Tau-12 cannot be used as capture and detection antibody at the same time, while all other total tau and tau isoform-specific antibodies in the panel are suitable to detect tau in hTg-Tau brain lysate. **b** All non-phospho-PTM modifications in the panel can be detected in hTg-Tau brain lysate. **c** All phospho-specific tau antibodies in the panel give signal from hTg-Tau brain lysate

In addition to the immunoblotting, we tested the specificity of the antibodies for tau by electrochemiluminescence ELISA using the same mouse brain lysates as for immunoblotting (Fig. 9). We chose this technique to assess the specificity of the antibodies to native tau, since we used denaturing conditions for immunoblotting. Moreover, ELISA can be performed in a sandwich format using a pair of antibodies, which may increase the specificity for tau. The electrochemiluminescence detection furthermore provides a quantitative and sensitive readout with a large linear range [13, 14].

We first biotinylated the antibodies in our panel and captured them on streptavidin-coated plates, then incubated the plates with cerebral lysates from hTg-Tau and Tau-KO mice followed by detection of the captured tau species with the pan-tau antibody Tau12. The capture antibody was linked to the sulfo-tag necessary to generate the electrochemiluminescent signal. We used biotinylated GAPDH antibody as a control for the background binding of tau on empty spots on the well and detected by sulfo-Tau12 antibody, and then subtracted this background signal from all other experimental data.

Amongst the pan-tau capture antibodies, HT7, BT2, Tau5, and Tau1 gave high signal in a similar range, which was 10^5^-fold higher in hTg-Tau compared to Tau-KO brain lysates. Dako-Tau, 1 N- and 2 N–isoform specific antibodies still showed a 10^4^-fold higher signal in hTg-Tau lysates compared to Tau-KO controls. Interestingly, when Tau12 was used both as capture and as detection antibody, we only detected background signal, suggesting that the Tau12 epitope is fully occupied in the capture step, preventing the binding of the sulfo-Tau12 detection antibody (Fig. 9a).

We then tested all other validated PTM-specific antibodies. The signal strength of the antibodies ranged between 10^4^ and 10^6^, whereas the background signal for all was around 10^2^ (Figs. 9b and c). These data suggest that all tau modifications tested are present in hTg-Tau mouse brain.

### Localization of tau species in human iPSC-derived neurons

**Fig. 10:**
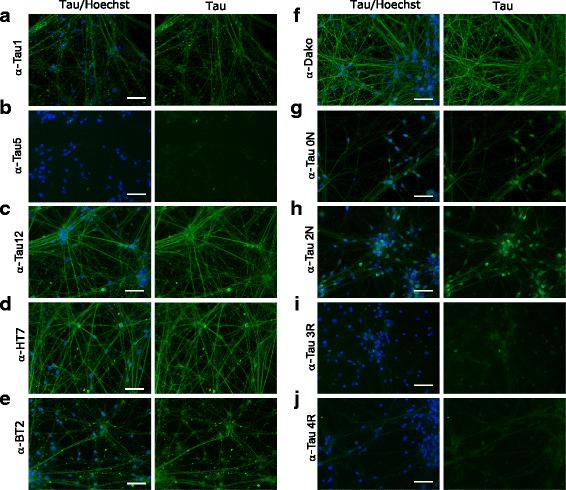
A panel of validated antibodies detects the subcellular distribution of tau in hiPSC-derived neurons. The panel of pan-tau (**a** - **f**, Tau1, Tau5, Tau12, HT7, BT2, and Dako) and isoform-specific (**g** - **j** 0 N, 2 N, 3R and 4R) tau antibodies were tested by immunofluorescence by using hiPSC-derived neurons. While no signal was observed for Tau5 (**b**), 3R (**i**) and 4R (**j**) antibodies, all other stainings detect tau in both cell bodies and neurites. Green: antibody staining, blue: Hoechst staining. Scale bars represent 100 μm

**Fig. 11:**
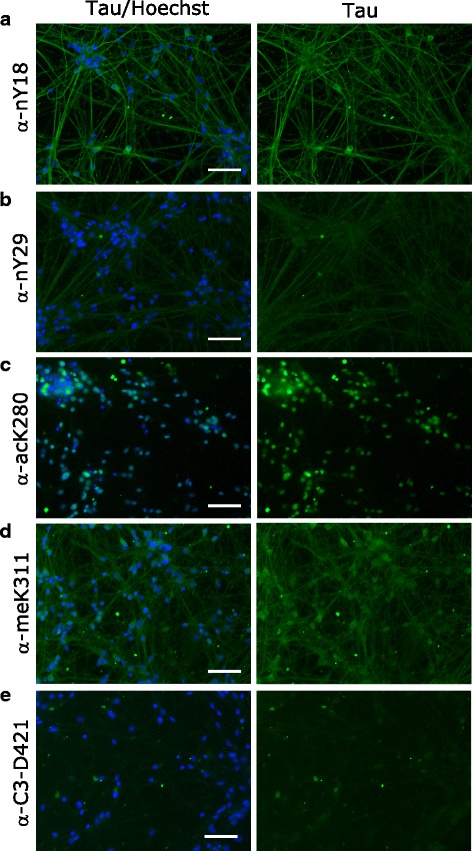
Non-phospho PTMs are present in hiPSC-derived neurons. nY18 (**a**) and meK311 (**d**) antibody staining reveals the presence of nitrated and dimethylated tau in both the cell bodies and neurites of hiPSC-derived neurons. acK280 staining shows nuclear localization (**c**), while no signal was obtained for the nY29 (**b**) and C3-D421 (**e**) modifications. Green: antibody staining, blue: Hoechst staining. Scale bars represent 100 μm

**Fig. 12:**
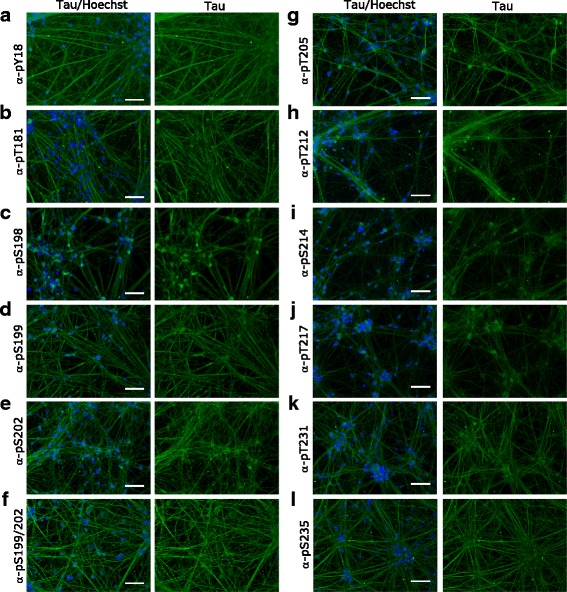
Immunofluorescence staining reveals a large number of tau phosphorylation events between amino acids Y18 and S235 in hiPSC-derived neurons. All phospho-tau antibodies in the panel showed staining in hiPSC-derived neuronal cultures. While most antibodies stained both the cytoplasm and the cell body (**a**, **c**, **d**, **e**, **f**, **g**, **h**, **i**, **j**, **k**), pT181 (**b**) and pS235 (**l**) signals were exclusively in the neurites. Green: antibody staining, blue: Hoechst staining. Scale bars represent 100 μm

**Fig. 13:**
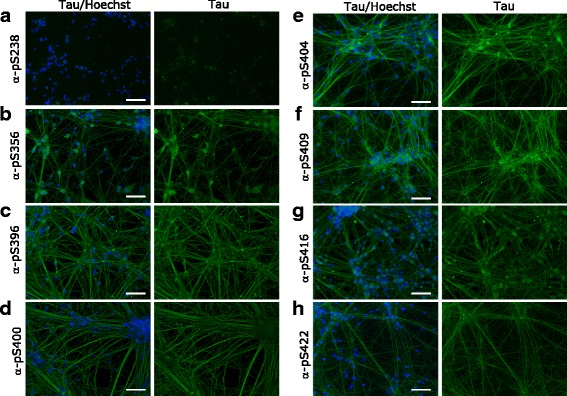
Immunofluorescence staining reveals a large number of tau phosphorylation events between amino acids S238 and S422 in hiPSC-derived neurons. All phospho-tau antibodies in the panel showed staining in hiPSC-derived neuronal cultures. While some antibodies stained both the cytoplasm and the cell body (**b**, **e**, **f**, **g**), pS238 signal was exclusively in the cell body (**a**). Antibodies against pS396 (**c**), pS400 (**d**), and pS422 (**h**) on the other hand preferentially stained neurites. Green: antibody staining, blue: Hoechst staining. Scale bars represent 100 μm

We next decided to use our panel of validated antibodies to determine whether PTMs alter the subcellular localization of tau in iPSC-derived neurons. While all tested pan-tau antibodies except Tau5 showed signal by immunofluorescence, the isoform specific antibodies 3R and 4R did also not work with this technique (Fig. 10). Furthermore, we could detect tau with all PTM-specific antibodies except C3-D421 (Figs. 11, 12 and 13). Interestingly, we observed differences in the localization of tau dependent on its modification. For example, tau phosphorylated at S198 localized both to cell body and neurites, whereas tau phosphorylated at T181 and acetylated at K280 mainly localized only to neurites or to cell body, respectively. In Table 2, we summarized the differential localization of tau species according to their presence in the cell body, in neurites or in both compartments.

**Table 2:** Localization of tau species in hiPSC-derived neurons

Name	Species	Company	Cat No.	Localization		Dilution
				Cell Body	Neurites	
Tau12	mouse	Biolegend	SIG-39416	**√**	**√**	1:100
Tau5	mouse	Abcam	ab80579	n.s.	n.s.	1:50
Tau1	mouse	Millipore	MAB3420	**√**	**√**	1:100
Dako	rabbit	Agilent Dako	A0024	**√**	**√**	1:200
BT2	mouse	Thermo Fisher	MN1010	**√**	**√**	1:100
HT7	mouse	Thermo Fisher	MN1000	**√**	**√**	1:100
Tau-0 N	mouse	Biolegend	823,801	**√**	**√**	1:50
Tau-2 N	mouse	Biolegend	816,801	**√**	**√**	1:50
Tau-3R	mouse	Millipore	05–803	n.s.	n.s.	1:50
Tau-4R	mouse	Millipore	05–804	n.s.	n.s.	1:50
nY18	mouse	Biolegend	829,701	**√**	**√**	1:50
nY29	mouse	Millipore	MAB2244	**√**	**√**	1:50
acK280	rabbit	Anaspec	AS-56077	**√**		1:50
meK311	mouse	Biolegend	MMS-5102	**√**	**√**	1:50
C3-D421	mouse	Millipore	36–017	n.s.	n.s.	1:25
pY18	mouse	Novusbio	NBP2–42402	**√**	**√**	1:100
pT181	rabbit	Thermo Fisher	701,530		**√**	1:100
pS198	rabbit	Abcam	ab79540	**√**	**√**	1:100
pS199	rabbit	Thermo Fisher	701,054	**√**	**√**	1:100
pS202	rabbit	Anaspec	AS-28017	**√**	**√**	1:100
pS199/202	rabbit	Thermo Fisher	44-768G	**√**	**√**	1:100
pT205	rabbit	Abcam	ab181206	**√**	**√**	1:100
pT212	rabbit	Abcam	ab51053	**√**	**√**	1:100
pS214	rabbit	Thermo Fisher	PA5–35762	**√**	**√**	1:100
pT217	rabbit	Thermo Fisher	44–744	**√**	**√**	1:100
pT231	rabbit	Thermo Fisher	701,056	**√**	**√**	1:100
pS235	rabbit	Thermo Fisher	PIPA535761		**√**	1:100
pS238	mouse	Abcam	ab128889	**√**		1:100
pS356	rabbit	Abcam	ab51036	**√**	**√**	1:100
pS396	rabbit	Thermo Fisher	44-752G		**√**	1:100
pS400	rabbit	Anaspec	AS-54978		**√**	1:100
pS404	rabbit	Thermo Fisher	44-758G	**√**	**√**	1:100
pS409	rabbit	Abcam	ab4861	**√**	**√**	1:100
pS416	rabbit	Abcam	ab119391	**√**	**√**	1:100
pS422	rabbit	Abcam	ab79415		**√**	1:25

Summarizes the differential localization of tau species stained with pan-tau or PTM-specific tau antibodies in hiPSC-derived neurons (Figs. 10, 11, 12 and 13). n.s.: no signal

## Discussion

Today, antibody reagents are recognized as one major source of reproducibility issues in biomedical research [7]. The so-called ‘reproducibility crisis’ has drawn public attention to problematic practices in pre-clinical research [15], and efforts to improve the robustness and reproducibility of primary research findings include guidelines for the thorough validation of antibody reagents [16].

While validation with knockout cells or animal tissues are becoming more common and are now also used by several major commercial antibody suppliers, PTM-specific antibodies need to clear yet another hurdle for specificity. In this study, we validated a panel of commercially available tau antibodies, which are sold to be specific to different PTM-modified versions of tau. Even though this panel covers all tau modifications that have been detected in human brain for which commercial antibodies are available, we would like to stress that our analysis does not cover all antibodies from all suppliers against these sites. Furthermore, we have not evaluated additional antibodies generated by academic researchers described in the literature.

Here we show that not all tau PTM-antibodies tested detect their specific modification site, and/or detect non-modified peptide in peptide array experiments. These findings are very concerning, since they will lead to the false-positive detection of PTMs and subsequent false conclusions drawn from experiments using these tools. Interestingly, validation of PTM-specific antibodies using a peptide array similar to the approach in this study is more commonly used for antibodies against histone PTMs [17]. We argue that this type of quality control is essential for all antibodies directed against modified proteins and should be done routinely, ideally by the manufacturer.

In our peptide arrays, we also observed that a PTM-specific antibody, although specific, may not detect its own site as other modifications in close vicinity may modify the epitope or block access for the antibody. Such false negative findings are especially important to consider for heavily modified proteins such as tau. Other approaches such as mass spectrometry or the use of smaller detection reagents such as camelid single-domain antibodies [18] may help to circumvent this issue. Antibodies that passed the specificity test were used in immunoblotting, ELISA and immunofluorescence experiments to evaluate their performance with denatured, native and fixed tau protein. In addition, we added total tau antibodies and isoform-specific antibodies to the panel at this point.

ELISA is a commonly used method for the quantitative assessment of tau and PTM-modified tau in biological samples, including patient CSF and plasma [19]. However, commercially available ELISA kits often do not specify which antibodies are used and only a small number of tau PTMs, all of which are phosphorylation, are covered. While previous academic studies have used antibody panels to detect the spectrum of tau PTMs in mouse models of AD [20, 21], our study significantly expands the spectrum of available methods by covering more PTMs and providing thorough validation of all antibodies used.

Only 4 out of 23 PTM-specific antibodies (meK311, C3-D421, pT217 and pS238) did not give a signal by immunoblotting using cerebral tissues of hTg-Tau mice, demonstrating that a large number of tau PTMs is present in this model. Interestingly, these four PTMs were detected by ELISA from the same hTg-Tau tissues, suggesting that they may be present but at levels below the detection limit for immunoblotting. Moreover, these antibodies may detect native form of tau better than its denatured form. Recently, a number of non-phospho-PTMs have been detected on tau and show alterations in AD brain tissue [21–26]. Our data suggest that these PTMs may be conserved in mouse models such as the hTg-Tau mice, making screens such as those identifying tau kinases [27, 28] feasible for other types of modifications.

iPSC-derived neurons are attractive systems to study human neurological disease, although modeling of neurodegeneration has been challenging due to the chronic and age-dependent phenotypes associated with diseases such as AD. Interestingly, we find here that most antibodies of our validated panel stain iPSC-derived neurons derived from a healthy control donor. Especially the presence of many phospho-tau epitopes is surprising in this context. While we cannot exclude that more intense staining could be observed in patient-derived cells, our findings suggest that the analysis of tau PTMs in such culture systems needs to be carefully controlled in order to draw conclusions about disease association.

Interestingly, our data suggest that PTMs may influence the subcellular localization of tau: Some antibodies preferentially stained the cell body, whereas others showed staining of tau in the neurites. It is possible that these patterns are driven by the localization of the kinases or other modifying enzymes, or that PTM-modified tau is preferentially transported to a specific subcellular region. Since PTMs are known to influence protein-protein interactions and may thus determine tau interaction partners, different tau PTMs may furthermore determine its function at any given point in time and space. While phosphorylation is strongly linked to tau’s function as a tubulin binding and stabilizing agent, it is possible that more recently identified tau PTMs regulate other functions of the protein such as its association with the ribonucleoproteome or its role in the postsynaptic targeting of Fyn in dendrites [29, 30].

## Conclusions

In summary, this study has created a platform to reliably and robustly detect changes in localization and abundance of post-translationally modified tau in health and disease. We believe that our data as well as the TauPTM online tool will be of high interest to the neuroscience community, and hope that our work will raise further awareness for the need for thorough validation of antibody-based tools and methods.

## Abbreviations

AD: Alzheimer’s disease
CSF: Cerebrospinal fluid
EC: Entorhinal cortex
ELISA: Enzyme-linked immunosorbent assay
hiPSC: Human induced pluripotent stem cell
hTg: Human transgenic
KO: Knock out
MAPT: Microtubule associated protein tau
PNS: Peripheral nervous system.
PTM: Post-translational modification
